# Clinicians as Leaders: Impact and Challenges

**DOI:** 10.12669/pjms.38.4.4918

**Published:** 2022

**Authors:** Rehan Nasir Khan, Ayesha Aziz, Nadeem Ahmed Siddiqui

**Affiliations:** 1 Rehan Nasir Khan, Aga Khan University Hospital, Karachi, Pakistan; 2 Ayesha Aziz, Aga Khan University Hospital, Karachi, Pakistan; 3 Nadeem Ahmed Siddiqui, Aga Khan University Hospital, Karachi, Pakistan

**Keywords:** Leadership, Medical education, Training

## Abstract

Healthcare has always been a complex system phenomenon which needs accountability from the leading clinical and management roles. Adequate and competent leadership is recognized as a driving point for a successful healthcare division. Medical professionals taking charge for quality improvement are well placed yet the style to deliver their leadership qualities bears a massive significance. While exceptional clinical prowess is of tremendous importance, harmonious teamwork, inefficiency reduction and patient communication and safety lead to noteworthy health management outcomes. Explores not explains the importance of leadership, qualities and styles of a leader, various leadership theories, the impact of leadership in medical education and the current issues related to medical leadership.

## INTRODUCTION

In a more literal sense, ‘Leadership’ refers to the skill and ability of an individual to either guide or influence a group of people. In the healthcare setting, leadership involves a complex interaction amongst different stakeholders, and is a vital trait in clinical, research and basic science practices.^1^ Leadership involves the ability of individuals to act as a guide with motivating influence and a coach for others, in order to create a social environment to solve a common task.^2^ In medical education, leadership involves interactions with group fellows to manage, develop and encourage the progression of service in order to provide and set the right direction. The effect of good leadership in medical education for the better academic and clinical outcomes is an emerging concept. It is thus important to properly understand the factors that influence leadership skills in the context of medical education. This report highlights the importance of clinical leadership, various leadership styles and the factors which influence the role of leadership in medical education.

### Why is Clinical Leadership Important?

It is a well-known fact that effective leadership bears paramount significance in the field of clinical sciences and the lack of this positive influence is analogous to organizational collapse.^1^-^3^ Clinical leadership is based upon the fundamental notion that the clinician will be able to put forward the rise in good communications and credibility. Clinical managers with a good grasp on health services may also make a well-informed decision that affects the allocation of available resources and design of the service.^4^ Literature demonstrates that a clinician’s role in the management of an institution is directly related to the overall effectiveness and better-quality parameters. Clinicians in senior management positions improve hospital and company operating outcomes substantially more than those with lower levels of clinician involvement. 5 It must be emphasized that the essential components of an optimal health care system, must be accountable of factors like system performance, achievement of health reform priorities, timely care delivery, system integrity, and reliability.^6^

### Leadership Qualities & Styles:

Clinical and administrative practitioners who collaborate to provide high-quality care must share and regard leadership equally.^3^ Healthcare workers, especially physicians wield tremendous power over the expenditure on healthcare. They hold the moral high ground of patient advocacy and enjoy a greater degree of autonomy as a result of their clinical training and expertise. Different approaches and styles of leadership can be classified, focusing on various organizational ideologies. Therefore, it is fundamental to understand leadership in medical education through the merits of its own classification.

Various qualities have been described in the literature as fundamental needs of a good leader. Such qualities include honesty, integrity, confidence, ability to inspire, commitment, passion, good communication skills, ability to make decisions, accountability, delegation, empowerment, creativity, innovation, empathy and above all, the ability to motivate others.^7^ Afore mentioned characteristics, the best style is usually a ‘flexible one’ that shifts from one style to another based on a combination of personality traits, modifiable behavioral patterns, and relevance to the situation.^8^

### The Impact of Leadership in Medical Education:

Recent literature indicates that leadership theories have been refined and modified over time, and no school of thought is simply inadequate. The form of leadership in operations necessitates a high level of precision, esteem, responsiveness, care, and domain competence may differ from that used in simple management-oriented portfolios, as one size does not fit all.^9^

After traditional education, leadership is the second most significant component which affects the learning of an individual. Effective leadership accounts for approximately a quarter of the student’s learning endeavors during the academic life.^10^ A clinician who is an effective leader achieves this by mentoring their learners and providing a proper academic environment to amplify innate teaching skills. Furthermore, it is essential for students and trainees to nurture leadership qualities, considering that they will be the future team leaders, and will be expected to effectively manage and coordinate large groups, be it in the clinical environment, the operating room, or even down to the research laboratory.

Thriving leadership strategies and successful organizational structure necessitate understanding learning ideas and settings, institutional regulations, and student population trends. Medical educators engage in a number of tasks that involve delivering quality teaching materials, creating a class curriculum, assessing and evaluating departments, educational programs and leading the whole team.^11^ To engage people, educational institutions must appreciate, encourage, and incentivize teachers and students. Careful consideration of the compatibility of faculty objectives with organizational goals is crucial. Another important factor influencing medical education, especially in current times, is ‘change and reform. It is essential that leader can understand this shift in change and lead with reform dynamics.^12^

Leaders who are involved in medical education must also understand and be able to operate across a variety of organizations and cultures with different systems and beliefs. As per education experts, it is critical that medical leaders have education as their first goal; however, this is more difficult in the healthcare sector owing to agenda disputes. This increases the pressure on leaders in medical education to strike a balance between the fast-expanding health care system, outstanding service provision for patients and communities, educational needs and expectations of the learners.^13^ Leaders must be conscious of these obstacles and must come up with appropriate and effective actions at the personal, collective and organizational levels. This enables people to influence change and enhance health systems via education, which is critical since it addresses essential concerns such as inter-professional education, diversity, and ingenuity.^14^

The style of leadership has also been shown to influence the commitment and quality of service. A study done in 43 hospitals of Sindh, showed that transformational and transactional leadership styles are positively associated with better commitment and quality of service.^15^

### Modern Health Care and Leadership:

Doctors are often expected to take on leadership roles in modern healthcare organizations. Aside from patient care, a modern clinician is often expected to make policy and financial management decisions. This is due to the fact that today’s healthcare system is dealing with a variety of demands that are constantly changing, as well as higher patient expectations and the rising cost of new interventions.^16^ As a consequence, modern physicians must make decisions for a larger patient group, use resources with excellent clinical competence, and execute service enhancements.^17^ To cope with the complicated reality of healthcare in a post-candor span, education leadership must personally guide our students to grow as competent and active leaders with reflective academics.

### The Current Challenges of Medical Education Leadership:

Attempting to explain the amount and manner in which clinical education institutions include components of organizational leadership is not intended to indicate that any such changes are unequivocally a welcome development. The available research, on the other hand, implies that this is a developing tendency, though one that is more prevalent in certain nations than others.^18^ A survey done in 336 medical students indicated that 147 (49.7%) students think leadership skills are important for clinicians and 145 (43.2%) students wish to have more leadership training in their medical schools.^19^

Clinical practice is an evolving phenomenon which requires medical fraternity to reinvent their leadership positions, particularly with the involvement of contributors and stakeholders, statutory officials and the general population, so as to yield the finest learning experiences for the students.^20^ The challenges in medical leadership have become rather evident in the recent times, including academic or clinical dichotomy, changing communication means like lack of interpersonal and face-to-face interactions and guidance, particularly in the COVID-19 pandemic era and an increase in administrative load. Apart from the escalating strain on the provision of medical care due to the global pandemic and public health needs, the provision of e-health care is unsatisfactory to many who prefer traditional medical care delivery. This preference for the old ways limits the application of effective transformations leading to ill-disposed orientation for medical professionals coming from non-traditional grounding, when top-management and lead positions are considered.^21^ Some of the commonly faced challenges and their impact are discussed in following section and Table-I.

**Table-I:** Table showing common challenges faced by clinician leader and their impact on healthcare outcomes.

Challenges	Impact
Unwelcoming peers and colleagues	Difficulty In implementation of reforms for improvement
Demand for leadership qualities	Negative impact on individual and organizational efficiency
Gender discrimination	Inadequate / Non utilization of potential workforce
Balance between incentives and reprimand	Complacency and non-motivation amongst team members
Lack of training as clinician leader	Inefficient delegation of work / responsibilities
Continually evolving healthcare demands	Stagnancy in provision of healthcare delivery and inability to progress with changing environment

### Leadership Demands:

An effective leader takes center stage of a group, influencing the actions of other members and interactions. This professional responsibility comes with great demands. One of the most critical factors in determining organizational effectiveness is the efficiency of leaders.^22^

### Gender Bias:

There is no scarcity of trained and well-qualified females for leadership positions. However, the qualities of a leader are still mainly based on the male stereotype. Moreover, managing career and home life may be difficult, limiting women’s opportunities for leadership roles. This inhibits scholarly breakthroughs, impedes economic growth, and interferes with bureaucracy, longevity, and utilization of talent.^23^

### Workforce Turbulence:

Owing to the fact that human variables are the seed of one of the biggest challenges for a leader, it is highly important for the employees to be reprimanded. Punishment type depends upon the personality and management style of a leader. Similarly, incentives associated with the ability to complete the assigned task can also help improve the efficiency of an individual and organization.

### Future Directions:

Considering the above-mentioned challenges, we emphasize the need for giving conscious efforts and establishing structured training for clinician as leaders and should be able to support them whenever needed. This support can be provided by enhancing their management skills, providing better mentorship and encourage peers and colleagues to provide support to function them as an efficient clinical leader. Undergraduate and postgraduate training must include the education and awareness about the importance of good clinical leader and institutes should be able to provide enough support if student wants to pursue their career as clinical managers. Further research is needed in some areas like gender discrimination and ability of female workforce to function as clinicians as well as leaders.

## CONCLUSION

The ultimate role of leadership in medical education is to prepare future practitioners for their inevitable role as team leads, irrespective of the career path they follow. A clinician or operating surgeon must be just as an effective group head, and influencing factor, as they are effective practitioners, in order to ensure the quality and flow of optimal patient care. Similarly, in educators and researchers, this trait is further essential to ensure ideal performance and motivation in projects. It must be emphasized, that inevitably good leadership, regardless of the style chosen, is essential for providing quality care, and continual improvement in healthcare standards. And it should thus be more encouraged throughout the initial education and training of healthcare professionals.

### Authors’ Contribution:

**RNK and** **NAS** were involved in concept of paper, literature search, manuscript writing, and proof reading of the manuscript. These authors are also accountable for the authenticity and integrity of this report.

**AA** was involved in manuscript writing and proof reading of this short report.
